# EBV‐positive B‐cell lymphomas and lymphoproliferative disorders: Review from the perspective of immune escape and immunodeficiency

**DOI:** 10.1002/cam4.4198

**Published:** 2021-08-13

**Authors:** Akira Satou, Shigeo Nakamura

**Affiliations:** ^1^ Department of Surgical Pathology Aichi Medical University Hospital Nagakute Japan; ^2^ Department of Pathology and Laboratory Medicine Nagoya University Hospital Nagoya Japan

**Keywords:** EBV‐positive lymphoma, immune escape, immunodeficiency, lymphoproliferative disorder, PD‐L1

## Abstract

**Background:**

Epstein‐Barr virus (EBV) is detected in a variety of B‐cell lymphomas (BCLs) and B‐cell lymphoproliferative disorders (B‐LPDs). Immunodeficiency has been considered to play a key role in the pathogenesis of these diseases. In addition, immune escape of tumor cells may also contribute to the development of EBV^+^ BCLs and B‐LPDs. The PD‐1/PD‐L1 pathway is particularly important for immune escape of tumor cells that contribute to development of lymphoma through suppression of cytotoxic T‐cell function. We now consider PD‐L1 immunohistochemistry (IHC) a very useful method for predicting whether tumor cells of lymphoid malignancies are characterized by the immune escape mechanism.

**Methods:**

We reviewed articles of EBV^+^ BCLs and B‐LPDs from the perspective of immune escape and immunodeficiency, particularly focusing on PD‐L1 IHC.

**Results:**

Based on PD‐L1 IHC, we consider that EBV^+^ BCL and B‐LPD can be classified into three types: “immunodeficiency”, “immune escape”, and “immunodeficiency + immune escape” type. The immunodeficiency type includes EBV^+^ diffuse large BCL (DLBCL) of the elderly, EBV^+^ sporadic Burkitt lymphoma, EBV^+^ mucocutaneous ulcer, and methotrexate (MTX)‐associated B‐LPD. The immune escape type includes EBV^+^ classic Hodgkin lymphoma (CHL) and EBV^+^ DLBCL of the young. The immunodeficiency + immune escape type includes CHL type MTX‐associated LPD and a minor subset of EBV^+^ DLBCL of the elderly.

**Conclusions:**

Recently, good results have been reported for immune check‐point inhibitors in treating lymphoma. Lymphomas and LPDs characterized by immune escape are regarded as good candidates for PD1/PD‐L1 blockade therapy. Therefore, from both the clinical and pathological perspective, we suggest that lymphoma diagnosis should be made considering immune escape and immunodeficiency.

## Graphical abstract

We now consider PD‐L1 immunohistochemistry (IHC) a very useful method for predicting whether tumor cells of lymphoid malignancies are characterized by the immune escape mechanism. Based on PD‐L1 IHC, we consider that EBV + BCL and B‐LPD can be classified into three types: “immunodeficiency,” “immune escape,” and “immunodeficiency + immune escape” type.

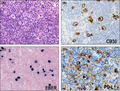

## 1 INTRODUCTION

According to the current WHO classification, Epstein–Barr virus (EBV) is detected in a variety of B‐cell lymphomas (BCLs) and lymphoproliferative disorders (B‐LPDs).^1^ Immunodeficiency has been considered as a key factor for the development of EBV^+^ BCLs and B‐LPDs.^2^, ^3^ Main causes of immunodeficiency are aging, infection of human immunodeficiency virus, and immunosuppressants used for patients after organ transplantation or with autoimmune disorders.^2^, ^3^

Immune escape of tumor cells may also play a key role in the pathogenesis of EBV^+^ BCLs and B‐LPDs. Particularly, the PD‐1/PD‐L1 axis is important for immune escape of tumor cells which contribute to the development of lymphomas via suppression of cytotoxic T‐cell function.^4^, ^5^, ^6^ Some previous studies reported that PD‐L1 expression on tumor cells predicts the worse outcomes for malignant lymphomas.^7^, ^8^, ^9^, ^10^, ^11^ These worse outcomes are considered to be caused by the immune escape mechanism of the lymphoma cells. Thus, we now consider PD‐L1 IHC a very useful method for predicting whether tumor cells of lymphoid malignancies are characterized by the immune escape mechanism; that is, lymphomas or LPDs expressing PD‐L1 on neoplastic cells are characterized by the immune escape mechanism.

Here, we review EBV^+^ BCLs and B‐LPDs from the perspective of immune escape and immunodeficiency. The EBV^+^ BCLs and B‐LPDs we describe in this article include EBV^+^ diffuse large BCL, not otherwise specified (DLBCL‐NOS), EBV^+^ sporadic Burkitt lymphoma (sBL), EBV^+^ classic Hodgkin lymphoma (CHL), EBV^+^ mucocutaneous ulcer (EBVMCU), methotrexate‐associated B‐LPD (MTX B‐LPD), and CHL type MTX‐associated LPD.

## 2 IMMUNE ESCAPE AND IMMUNODEFICENCY OF EBV^+^ BCLS AND B‐LPDS

Immune escape is a key factor of lymphomagenesis. Cancer cells have a variety of mechanisms to escape from immunosurveillance.^12^, ^13^ For example, they recruit regulatory T cells (Tregs) and myeloid‐derived suppressor cells (MDSCs). These two are major types of immunosuppressive regulatory immune cells. Activated Tregs attenuate the function of tumor‐specific T lymphocytes by secreting IL‐10 and TGF‐β, which are immunosuppressive cytokines, and by expressing PD‐1, CTLA‐4, and PD‐L1. MDSCs are a heterogeneous group of immature myeloid cells and myeloid progenitor cells. MDSCs possess strong immunosuppressive activities regulating the functions of other types of immune cells, such as macrophages, T lymphocytes, dendritic cells, macrophages, and natural killer (NK) cells. Another mechanism is the production of immunosuppressive cytokines, including VEGF, TGF‐β, galectin, or IDO, by cancer cells. They also escape from immunosurveillance by expressing immune checkpoints, for example, PD‐L1 and CTLA‐4. Overall, the expression of PD‐L1 is considered to be the dominant mechanism.

PD‐L1 expression on cancer cells is caused by two general mechanisms, known as innate and adaptive immune resistance.^14^, ^15^ The former mechanism causes constitutive expression of PD‐L1 via aberrant signaling pathways or genetic alterations of *PD*‐*L1*. For example, the aberrant signaling pathways which induce PD‐L1 expression are driven by PTEN losses in glioblastoma, EGFR mutations in lung carcinoma, and BRAF V600E mutations in malignant melanoma.^16^, ^17^, ^18^, ^19^ In addition, genetic alterations in *PD*‐*L1*, which induce PD‐L1 expression have been reported. Alteration of the 9p24.1/*PD*‐*L1*/*PD*‐*L2* has been reported in malignant lymphoma and gastric adenocarcinoma.^20^, ^21^ Another important genetic alteration in *PD*‐*L1* is 3’‐UTR disruption. Kataoka et al.^22^ revealed that PD‐L1 overexpression is caused by *PD*‐*L1* 3’‐UTR disruption in multiple cancers. Another mechanism that causes PD‐L1 overexpression is gene fusion between CIITA and upstream of PD‐L1, which is commonly detected in primary mediastinal large B‐cell lymphoma.^23^ The *CIITA–PD‐L1* fusion places PD‐L1 under the transcriptional control of the CIITA promoter and drives PD‐L1 overexpression. Among the EBV^+^ BCLs and B‐LPDs, almost all EBV^+^ CHLs and a subset of EBV^+^ DLBCLs have alterations in *PD*‐*L1* and *PD*‐*L2*.^21^, ^24^ Another mechanism that induces PD‐L1 expression on tumor cells is adaptive immune resistance. The overexpression of PD‐L1 is driven by interactions with cytokines, particularly IFN‐γ produced by immune cells in the tumor microenvironment (TME).^25^ IFN‐γ is secreted by activated CD8^+^ T cells, activated Th1‐type CD4^+^ T cells, and NK cells. The cancer cells show adaptive responses against IFN‐γ, resulting in PD‐L1 induction and avoiding attacks by inflammatory immune microenvironment.

The immunodeficiency of EBV^+^ BCLs and B‐LPDs reviewed in this article is caused by aging or MTX use for autoimmune diseases. Immunosenescence is a gradual decline in immune functions brought on by the aging process. Aging promotes an imbalance between inflammatory and inflammatory‐neutralizing processes. This imbalance cause the low‐grade chronic pro‐inflammatory state,^26^ which induces free radicals that cause DNA damage, resulting in carcinogenesis, including lymphomagenesis.^27^ Immunosenescence also causes a decrease in the number of total T cells, including cytotoxic CD8^+^ and helper CD4^+^ cells, and a gradual decline in the T‐cell antigenic repertoire.^28^, ^29^ Thus, the number of CD8^+^ EBV‐specific T cells is reduced. MTX is a cytoreductive folate analog drug commonly used to treat autoimmune diseases, particularly rheumatoid arthritis (RA).^30^ Multiple mechanisms are considered to be affected in the treatment of RA.^31^ For example, MTX reduces cell proliferation and increases T‐cell apoptosis. Another mechanism is the reduction of the cytokine production induced by T‐cell activation. Therefore, the immunodeficient status of patients due to aging and MTX use suppresses the function of CD8^+^ EBV‐specific T cells that regulate EBV^+^ cell proliferation.^32^ This immune dysregulation in the surveillance for EBV is presumed to result in the development of EBV^+^ BCL and B‐LPD.

## 3 DISEASE OVERVIEW: EBV^+^ BCLs and B‐LPDs

### 3.1 EBV^+^ DLBCL‐NOS

EBV^+^ DLBCL‐NOS was first described as age‐related EBV‐associated LPD with no evidence of underlying immunodeficiency in 2003.^1^ In the previous version of WHO classification, this disease was documented as EBV^+^ DLBCL of the elderly and is now changed to EBV^+^ DLBCL‐NOS in the 2017 WHO classification. The age restriction has been deleted because the disease may arise in a wide age range.^33^ However, the majority of EBV^+^ DLBCL‐NOS occur in individuals older than 50, and the percentage of the EBV^+^ group for all DLBCL‐NOS cases increases with age, peaking in the 80s.^34^, ^35^, ^36^, ^37^, ^38^ Therefore, immunosenescence is suggested to play an important role in the development of EBV^+^ DLBCL of the elderly.

**FIGURE 1:**
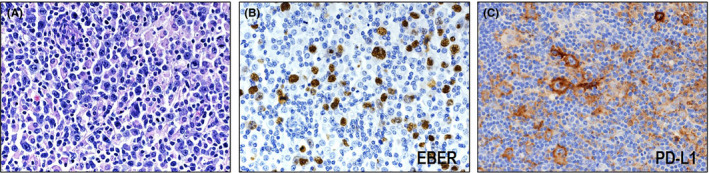
Histological and immunohistochemical features of EBV‐positive diffuse large B‐cell lymphoma (DLBCL) of the elderly. (A) Histologically, a polymorphic pattern is observed in this case (HE ×400). (B) The tumor cells show positive staining for EBER (×400). (C) PD‐L1 expression was detected in 11% of EBV^+^ DLBCL of the elderly (×400)

In a Japanese cohort, Kiyasu et al.^7^ recently reported that 16% (14/90) of patients with EBV^+^ DLBCL‐NOS expressed PD‐L1. In addition, Takahara et al.^9^ recently reported that 11% (6/57) of patients with EBV^+^ DLBCL‐NOS present with positive staining for PD‐L1 (Figure 1A–C). These two cohorts consisted mainly of old patients. Kataoka et al.^24^ reported that 19% (5/27) of EBV^+^ DLBCL‐NOS cases involved *PD*‐*L1*/*PD*‐*L2* somatic alterations. The frequency of *PD*‐*L1*/*PD*‐*L2* aberrations in EBV^+^ DLBCL‐NOS was much higher than the frequency in EBV^−^ DLBCL‐NOS. This frequency was comparable to the PD‐L1‐positive rate in EBV^+^ DLBCL‐NOS, suggesting that the PD‐L1 overexpression in this disease is mainly due to *PD*‐*L1*‐involving genetic alterations. They also found that structural variations (SVs) in *PD*‐*L2* were found exclusively in BCLs, whereas SVs involving PD‐L1 were detected in a variety of solid cancers, T/NK‐cell lymphomas, and BCLs. These results imply that immune escape may be another factor of lymphomagenesis in a minor subset of EBV^+^ DLBCL of the elderly. Takahara et al.^9^ further highlighted that patients with PD‐L1^+^ EBV^+^ DLBCL‐NOS were characterized by a poorer prognosis than PD‐L1^−^ cases. Based on this finding, we suggest that, along with immunosenescence, immune escape of tumor cells plays a key role in the lymphomagenesis of PD‐L1^+^ cases and contributes to their poorer prognosis. On the other hand, compared to immune escape, immunosenescence deeply affect the pathogenesis of PD‐L1^−^ cases, which account for the majority of EBV^+^ DLBCL of the elderly.

The frequency of PD‐L1 positivity was significantly higher in EBV^+^ LBCL of the young (≤45 years) than EBV^+^ DLBCL of the elderly. Nicolae et al.^33^ revealed that 77% (24/31) of EBV^+^ LBCL of the young cases expressed PD‐L1. They also revealed that all of the young patients present with nodal lesions, which comprise three morphological patterns: T‐cell/histiocyte‐rich large B‐cell lymphoma (THRBCL)‐like, gray zone lymphoma (GZL), and DLBCL‐NOS. Among the three, the THRBCL‐like pattern presented the highest frequency of PD‐L1 positivity (95%, 20/21). Furthermore, they highlighted that EBV^+^ LBCL of the young had significantly better overall survival (OS) than EBV^+^ DLBCL of the elderly (5‐year OS probability: 89.0% and 24.4%, respectively). In particular, young patients with the THRBCL‐like pattern had a superior OS curve compared to the other two patterns. Based on their findings, we speculate that immune escape of neoplastic cells promoted by PD‐L1 expression play a key role in the development of EBV^+^ LBCL of the young, whereas immunosenescence plays a far lesser role in the pathogenesis because young patients have potent immune responses. The good prognosis of young patients can be explained by the dependence of tumor cells on immune escape. In immune potent patients, when the lymphoma cells are eliminated by chemotherapy, there are no more factors that can lead to lymphomagenesis.

We consider that EBV^+^ DLBCL of the elderly and EBV^+^ DLBCL of the young should be regarded as different pathogenic types. The former is categorized as immunosenescent type, and the latter as immune escape type. A minor subset of EBV^+^ DLBCL of the elderly cases express PD‐L1, and they are considered to be categorized as immunosenescent + immune escape type.

### 3.2 EBV^+^ CHL

CHL is a malignant tumor derived from B cell and is composed of Hodgkin and Reed–Sternberg (HRS) cells on a background containing extensive non‐neoplastic immune cells.^1^, ^39^ Based on the characteristics of infiltrating immune cells and morphological findings, CHL is divided into four histological subtypes: nodular sclerosis (NS), mixed cellularity (MC), lymphocyte‐rich (LR), and lymphocyte‐depleted (LD).^1^ The frequencies of EBV positivity on HRS cells varies among the subtypes, and are higher in MC type (75%) and LD type (75%) and lower in NS type (10%–25%) and LR type (variable).^1^, ^39^ Overall, HRS cells are EBV‐positive in about 40% of CHL cases.^40^

**FIGURE 2:**
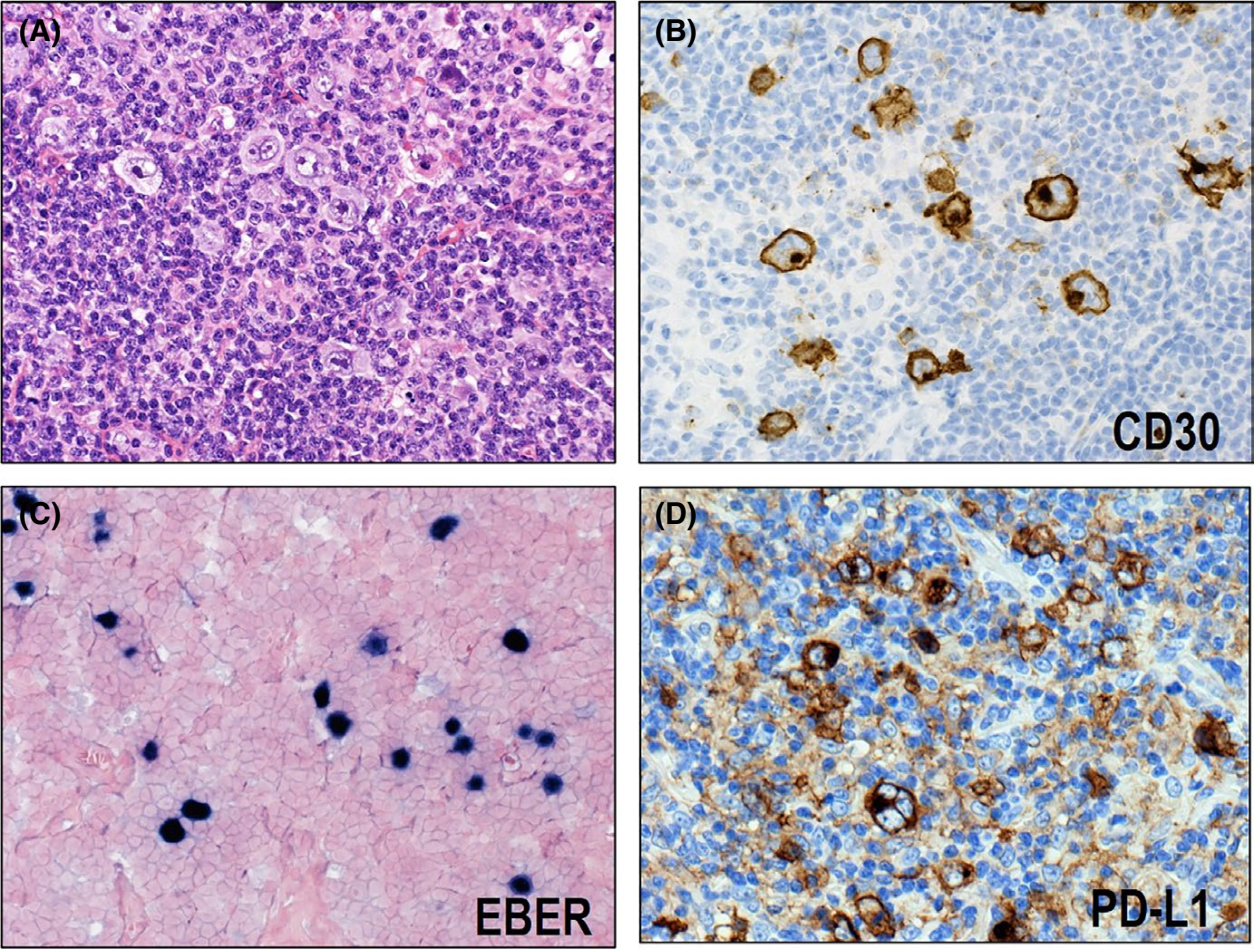
Histological and immunohistochemical features of EBV‐positive mixed cellularity classic Hodgkin lymphoma (CHL). (A) Hodgkin and Reed–Sternberg (HRS) cells proliferate on a background containing extensive non‐neoplastic immune cells (HE ×400). (B) The HRS cells are CD30‐positive (×400) and (C) EBER‐positive (×400). (D) All of the EBV^+^ CHLs show positive staining for PD‐L1 (×400)

CHL is now considered as a representative immune escape‐type lymphoma. Green et al.^41^ identified *PD*‐*L1* and *PD*‐*L2* as key targets of 9p24.1 copy gain in cell lines of CHL and HRS cells captured by laser microdissection. They also reported, using quantitative immunohistochemical methods, that PD‐L1 expression level correlated with *PD*‐*L1* copy number. Besides *PD*‐*L1* and *PD*‐*L2*, *JAK2* is also included in the broader 9p24.1 amplification region. *JAK2* copy gain enhances JAK2 expression and JAK/STAT signaling, which leads to the expression of PD‐L1. They also revealed that EBV infection induces the expression of PD‐L1 in CHLs. This increased expression of PD‐L1 is mediated by LMP1 through the JAK/STAT and AP‐1 pathways. Roemer et al.^21^ performed FISH in 108 samples from CHL cases to evaluate the alterations of *PD*‐*L1* and *PD*‐*L2*. They found that 99% (107/108) of CHLs had *PD*‐*L1* and *PD*‐*L2* aberrations, including polysomy (*n* = 5), copy gain (*n* = 61), and amplification (*n* = 39). They also identified that PD‐L1 expression was associated with relative genetic aberrations. In this series of CHL cases, the EBV^+^ and EBV^−^ CHLs had similar distribution of genetic alterations. However, HRS cells in EBV^+^ CHLs had a higher percentage and stronger intensity of PD‐L1‐positive staining, which indicates PD‐L1 expression is further induced by EBV infection. Sakakibara et al.^42^ also confirmed in their series that all of the EBV^+^ CHLs, including one NS type and nine MC type, stained positive for PD‐L1 in IHC (Figure 2A–D). Therefore, immune escape is suggested to be strongly associated with the pathogenesis of EBV^+^ CHL.

The TME of CHL, including both EBV^+^ and EBV^−^ cases, has been thoroughly analyzed and its contributions to the immune escape mechanism of CHL highlighted. Previous studies have highlighted that CD4^+^ T cells are abundant in the TME of CHL.^43^, ^44^, ^45^, ^46^ Aoki et al.^45^ recently reported a single‐cell transcriptome analysis identifying a novel subset of T cells in the TME of CHL, the LAG3^+^ T‐cell population. LAG3^+^ T cells have Treg‐like features and contribute to the immune escape phenotype. Carey et al.^44^ found that the majority of PD‐L1^+^ cells in the TME of CHL are tumor‐associated macrophages (TAMs). These PD‐L1^+^ TAMs regionally colocalize with HRS cells, suggesting that the elevated expression level of PD‐L1 on TAMs is in response to cytokine production, particularly IFN‐γ, by HRS cells. Consequently, the total amount of PD‐L1 in the vicinity of the HRS cells is increased, and the immune escape mechanism of CHL may be enhanced.

### 3.3 EBV^+^ sBL

BL is a B‐cell neoplasm with highly aggressive behavior. BL is classified into three clinical variants: endemic, sporadic, and immunodeficiency‐associated.^1^ Endemic BL (eBL) occurs in equatorial Africa and Papua New Guinea, which are also endemic for malaria.^47^ Sporadic BL (sBL) is seen worldwide, and immunodeficiency‐associated BL most commonly occurs in HIV patients.^48^, ^49^ EBV is detected in >95% of eBL, 20%–30% of sBL, and 25%–40% of immunodeficiency‐associated BL.^48^, ^50^ In Western Europe and North America, sBL occurs mainly in children and young. sBL accounts for 30%–50% of childhood lymphomas in these counties.^51^

Satou et al.^50^ previously reported that 22% (33/150) of sporadic BL cases in Japan were infected with EBV. Comparing the clinicopathological characteristics of EBV^+^ and EBV^−^ BL, they found that EBV^+^ BL had a significantly higher age distribution. The difference in age distribution can be explained by the fact that the most of pediatric cases were EBV^−^. They speculated that immunodeficiency plays an important role in the development of EBV^+^ sBL. In old patients with EBV^+^ sBL, they suggested that the immunodeficiency was caused by aging. Therefore, EBV^+^ sBL arising in old patients may have a characteristic of age‐related LPD. In contrast, the immunodeficient condition of young patients with EBV^+^ sBL is caused by an unknown mechanism.

Chen et al.^52^ assessed the PD‐L1 expression in a series of EBV^+^ lymphomas by IHC, but none of the seven cases of EBV^+^ BL was positive for PD‐L1 on tumor cells. In our cohort of EBV^+^ sBL, five cases tested for PD‐L1 expression were negative for PD‐L1 (unpublished data). These results suggest that immune escape play a lesser role in the pathogenesis of EBV^+^ sBL, whereas immunodeficiency has a key function in its development.

### 3.4 EBVMCU

Dojcinov et al. first reported EBVMCU in 2010, and it was newly categorized as a provisional entity in the 2017 WHO classification.^1^, ^53^ This disease presents as sharply circumscribed, isolated lesions confined to the oral mucosa, skin, and gastrointestinal tract. There is no evidence of systemic involvement.^53^ EBVMCU occurs in individuals of varying immunodeficient status (older age, immunosuppressant drug treatment, treated lymphoma, HIV infection, and primary immunodeficiencies) and is now considered as a specific type of immunodeficiency‐associated LPD.^53^, ^54^, ^55^, ^56^, ^57^, ^58^, ^59^ Patients with EBVMCU typically have an indolent, self‐limited course with spontaneous remission (SR) or complete remission (CR) after reduced immunosuppression. Thus, to distinguish EBVMCU from EBV^+^ DLBCL is important because clinical approach can be different. Recently, Ikeda et al.^54^ performed a clinical and pathological study of 34 Japanese patients with EBVMCU; causes of immunosuppression in these 34 patients were MTX use (*n* = 30), hydroxycarbamide use (*n* = 1), and aging (*n* = 3). Therefore, in the Japanese cohort, a majority of the EBVMCUs may have arisen as a subtype of EBV^+^ MTX B‐LPD, which is described below. EBVMCU was histologically classified into four subtypes: polymorphous; large cell‐rich; CHL‐like; and mucosa‐associated lymphoid tissue lymphoma‐like. The occurrence of *IGH* rearrangement was not significantly different between EBVMCU and EBV^+^ DLBCL, or EBVMCU and EBV^−^ DLBCL. Thus, they concluded that EBVMCU was difficult to distinguish from EBV^+^ DLBCL based on histological and genetical findings, and clinical information is necessary for the accurate diagnosis of EBVMCU.

**FIGURE 3:**
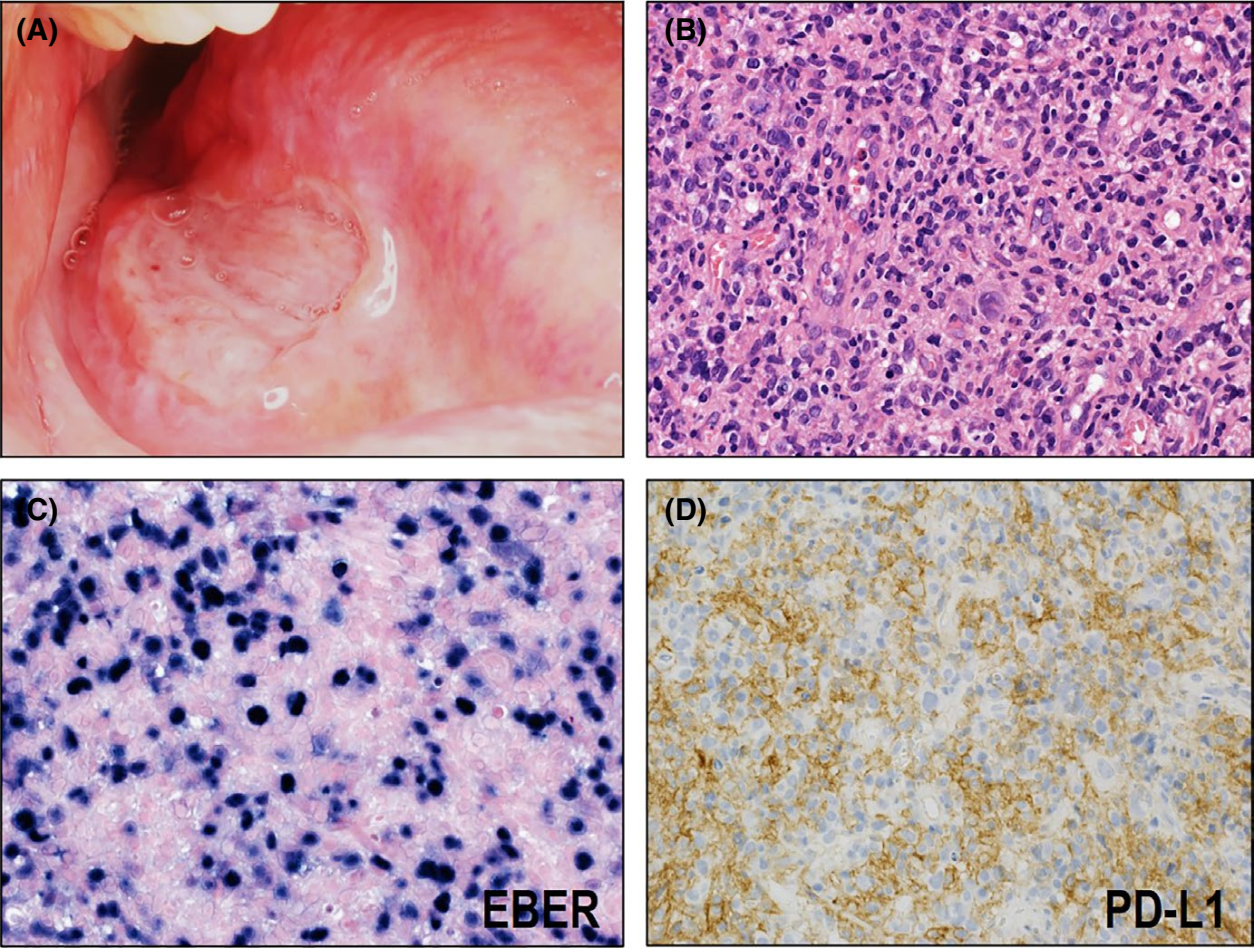
Macroscopic, histological, and immunohistochemical features of EBV‐positive mucocutaneous ulcer (EBVMCU). (A) EBVMCU macroscopically shows a sharply circumscribed mucosal ulcer. (B) Histologically, EBVMCU shows infiltration of intermediate to large atypical lymphoid cells with polymorphous background (HE ×400). (C) EBER is detected in the large atypical lymphoid cells (×400). (D) Most of the cases are negative for PD‐L1 on tumor cells (×400)

Daroontum and Satou et al. previously assessed PD‐L1 expression in 25 EBVMCU cases.^55^, ^56^ None of them presented positive staining for PD‐L1, except for one case (Figure 3A–D). Therefore, immune evasion mechanism may not associate with most EBVMCU cases. This result is expected because EBVMCU is considered as a specific type of immunodeficiency‐associated LPD, and immunodeficiency due to various conditions is regarded as the main cause of the pathogenesis of this disease.

### 3.5 MTX B‐LPD and CHL type MTX‐associated LPD

MTX is an antirheumatic drug widely used to treat autoimmune diseases, particularly rheumatoid arthritis (RA).^30^ Although MTX has an excellent inhibitory effect against articular destruction, it increases the risk of LPD.^60^ According to the 2017WHO classification, MTX‐associated LPD is categorized as “other immunodeficiency‐associated LPDs.”^1^ The iatrogenic immunodeficient status due to MTX use contributes to the development of various types of LPD, including reactive lymphoid hyperplasia, low‐grade BCL, polymorphic B‐LPD, DLBCL, peripheral T‐cell lymphoma, and CHL.^61^, ^62^, ^63^ Among these, DLBCL type occurs most frequently, followed by CHL and polymorphic B‐LPD. Each of these types includes both EBV^+^ and EBV^−^ cases.

MTX‐associated LPD is unique in that they may present with spontaneous regression after the MTX cessation.^61^, ^62^, ^63^ This phenomenon supports the theory that the disease is caused as a result of immunosuppression induced by MTX. In particular, EBV^+^ cases have a higher frequency of SR than EBV^−^ cases.^61^, ^62^ On the other hand, CHL type MTX‐associated LPD is characterized by a lower frequency of SR after MTX cessation.^64^ Gion et al.^64^ reported that most patients with CHL type could not be cured by MTX cessation alone and required additional chemotherapy. They also revealed that CHL type had an inferior progression‐free survival curve compared to DLBCL type. As many studies of MTX‐associated LPD have been performed, clinicopathological aspects of the disease have been gradually highlighted.

**FIGURE 4:**
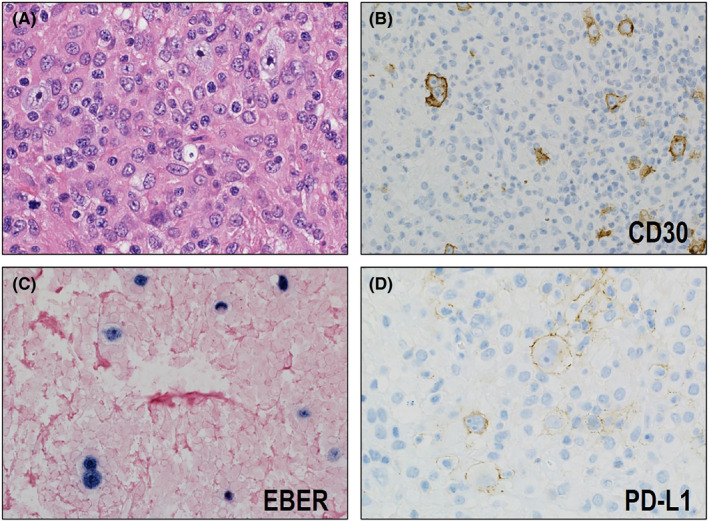
Histological and immunohistochemical features of classic Hodgkin lymphoma (CHL)‐type methotrexate (MTX)‐associated lymphoproliferative disorder (LPD). (A) Hodgkin and Reed–Sternberg (HRS) cells proliferate on a background containing extensive non‐neoplastic immune cells (HE ×400). (B) The HRS cells are CD30‐positive (×400) and (C) EBER‐positive (×400). (D) Most of the CHL‐type MTX‐associated LPDs stain positive for PD‐L1 (×400)

The mechanism underlying the differences in clinical behavior among the types of MTX‐associated LPDs remain to be elucidated. Kohno et al.^65^ recently reported interesting findings that may account for the clinical characteristics of CHL type MTX‐associated LPD. They performed a PD‐L1 immunohistochemical assessment in a series of MTX‐associated LPDs, including CHL type (*n* = 9), DLBCL type (*n* = 15), and polymorphic B‐LPD type (*n* = 21). Notably, PD‐L1 expression on neoplastic cells was found exclusively in CHL type (89%, 8/9) (Figure 4A–D). Fluorescence in situ analysis was performed to assess the *PD*‐*L1* copy number status for two cases of CHL type, detecting PD‐L1 gene amplification in one of them. These results imply that, in addition to immunodeficiency due to MTX use, immune escape mechanism contribute to the development of CHL type MTX‐associated LPD. This pathogenic model may account for the lower frequency of SR and frequent demand for additional chemotherapy. Namely, when the immune system of patients recovers after MTX cessation, the antitumor responses of cytotoxic T cells may be regained. This recovery of the antitumor response leads to SR of the lesions. However, the immune escape mechanism of tumor cells in CHL type still remain after MTX cessation, and they evade the patient's immune surveillance. Therefore, the frequency of SR is low in CHL type and additional chemotherapy is required to cure the disease.

## 4 CONCLUSIONS

We reviewed EBV^+^ BCL and B‐LPDs from the perspective of immune escape and immunodeficiency. We used PD‐L1 IHC as an indicator of whether tumor cells harbor an immune escape mechanism, and classified EBV^+^ BCL and B‐LPD into three types: immunodeficiency, immune escape, and immunodeficiency + immune escape. The immunodeficiency type is PD‐L1‐negative and considered to be due to immunodeficiency caused by aging or MTX use. The immune escape type is PD‐L1‐positive and less associated with immunodeficiency. The immunodeficiency + immune escape type is PD‐L1‐positive and considered to be associated with immunodeficiency.

Recently, good results of immune checkpoint inhibitors in treating lymphoma have been reported.^66^, ^67^, ^68^, ^69^ Lymphomas and LPDs characterized by immune escape of the tumor cells are regarded as good candidates for PD1/PD‐L1 blockade therapy. For example, CHL, a representative immune escape‐type lymphoma, shows a good response to PD‐L1 blockade.^66^, ^70^, ^71^ Therefore, from both the clinical and pathological perspective, we suggest that lymphoma diagnosis should be made considering immune escape and immunodeficiency.

## CONFLICT OF INTERESTS

The authors declare no competing financial interests.

## Data Availability

Data sharing is not applicable to this article as no datasets were generated or analyzed during this study.
